# Asymptomatic Bacteriuria in Clinical Urological Practice: Preoperative Control of Bacteriuria and Management of Recurrent UTI

**DOI:** 10.3390/pathogens5010004

**Published:** 2016-01-05

**Authors:** Tommaso Cai, Sandra Mazzoli, Paolo Lanzafame, Patrizio Caciagli, Gianni Malossini, Gabriella Nesi, Florian M. E. Wagenlehner, Bela Köves, Robert Pickard, Magnus Grabe, Truls E. Bjerklund Johansen, Riccardo Bartoletti

**Affiliations:** 1 Department of Urology, Santa Chiara Regional Hospital, Largo Medaglie d’Oro 9, 38123 Trento, Italy; gianni.malossini@apss.tn.it; 2 Sexually Transmitted Disease Centre, Santa Maria Annunziata Hospital, 50012 Florence, Italy; mazzoli49@yahoo.com; 3 Department of Microbiology, Santa Chiara Regional Hospital, 38123 Trento, Italy; paolo.lanzafame@apss.tn.it; 4 Department of Laboratory Medicine, Santa Chiara Regional Hospital, 38123 Trento, Italy; patrizio.caciagli@apss.tn.it; 5 Division of Pathological Anatomy, Department of Critical Care Medicine and Surgery, University of Florence, 50100 Florence, Italy; gabriella.nesi@unifi.it; 6 Klinik und Poliklinik für Urologie, Kinderurologie und Andrologie, Universitätsklinikum Giessen und Marburg GmbH, Justus-Liebig-Universität Giessen, 35390 Giessen, Germany; wagenlehner@aol.com; 7 Department of Urology, South-Pest Hospital, 1051 Budapest, Hungary; bkoves@gmail.com; 8 Institute of Cellular Medicine, Newcastle University, Newcastle upon Tyne NE14XE, UK; robert.pickard@newcastle.ac.uk; 9 Department of Urology, Skåne University Hospital, University of Lund, S-20502 Malmö, Sweden; magnus@grabe.se; 10 Department of Urology, Oslo University Hospital, 0271 Oslo, Norway; trulsebj@frisurf.no; 11 Department of Urology, University of Florence, 50012 Florence, Italy; riccardo.bartoletti@unifi.it

**Keywords:** asymptomatic bacteriuria, antibacterial agents, prophylaxis, pre-operative prophylaxis, antibiotic susceptibility, UTI

## Abstract

Asymptomatic bacteriuria (ABU) is a common clinical condition that often leads to unnecessary antimicrobial use. The reduction of antibiotic overuse for ABU is consequently an important issue for antimicrobial stewardship and to reduce the emergence of multidrug resistant strains. There are two issues in everyday urological practice that require special attention: the role of ABU in pre-operative prophylaxis and in women affected by recurrent urinary tract infections (rUTIs). Nowadays, this is the time to think over our practice and change our way of thinking. Here, we aimed to summarize the current literature knowledge in terms of ABU management in patients undergoing urological surgery and in patients with rUTIs. In the last years, the approach to patient with ABU has changed totally. Prior to all surgical procedures that do not enter the urinary tract, ABU is generally not considered as a risk factor, and screening and treatment are not considered necessary. On the other hand, in the case of all procedures entering the urinary tract, ABU should be treated in line with the results of a urine culture obtained before the procedure. In patients affected by rUTIs, ABU can even have a protective role in preventing symptomatic recurrence, particularly when *Enterococcus faecalis* (*E. faecalis*) has been isolated.

## 1. Introduction and Background

Asymptomatic bacteriuria (ABU), defined as the presence of bacteria in the urine of an individual without signs or symptoms of a urinary tract infection [1], is generally present in 3% to 5% of young women and is more common in patients with diabetes and elderly persons [2]. ABU treatment is generally recommended only in pregnant women and at the pre-operative evaluation before surgical procedures [2]. Regardless of these recommendations, overuse of antibiotics in ABU treatment is common. In fact, about one-third of ABU were over-treated against guidelines, with important negative consequences on public health [3,4,5]. For many years, the inappropriate use of antibiotics has been recognized as a major problem, leading to higher healthcare costs as well as increased antimicrobial resistance through the selection and spread of drug-resistant microorganisms with severe consequences on patient health [6,7]. The optimization of antibiotic usage would not only prevent increased resistance, but would also limit all related costs. There are two main conditions in urological and everyday clinical practice that require specific considerations: the role of ABU in pre-operative prophylaxis and in women affected by recurrent urinary tract infections (rUTIs). Even if prophylactic antibiotics are effective in a wide range of surgical procedures and have contributed substantially to reduce postoperative infectious complications, the uncorrected use of antibiotics in urological surgery and an extended duration of antibiotic administration after surgical procedure are correlated with important consequences, such as the appearance of multi-resistant organisms, including strains resistant to newer agents, worsened clinical outcome and increased treatment costs [4,8,9]. Even though the European Association of Urology (EAU) guidelines on *urological infections* are easy to read and consult for the urologist [10], the compliance with EAU guidelines in urological surgery is not optimal [11]. In particular, the role of ABU before urological surgery is not totally understood. On the other hand, the role of ABU in the management of women with rUTIs is not totally clear, either. Sometimes, in everyday clinical practice, we note that young sexually active women affected by recurrent UTI showed, after a course of antibiotic treatment, an asymptomatic period associated with sterile urine and then develop an episode of ABU [12]. In the majority of cases, even if it is not recommended, ABU is treated with poor results and with a high risk of selecting multidrug resistance [13]. Recently, Cai *et al.* even demonstrated the protective effect of spontaneously developed ABU in women with rUTIs and without identified risk factors [12]. However, some authors stated that occasionally the eradication of a strain considered the causative agent of recurrent episodes of UTI may be justified [14]. From this background, two important questions are:

- What is the role of ABU treatment prior to surgery?
- What is the role of ABU treatment in women with recurrent urinary tract infections?

## 2. Results from the Current Literature

### 2.1. The Role of Asymptomatic Bacteriuria Prior to Surgery: To Treat or Not To Treat?

The EAU guidelines on urological infections suggested that bacteriuria is a definite risk factor in procedures entering the urinary tract and breaching the mucosa (endoscopic urological surgery), and in this sense it should be treated [14]. Moreover, they suggested that a urine culture must therefore be taken prior to such interventions, and in the case of ABU, pre-operative treatment should be given [15]. These recommendations are very important for the clinical practice because they highlight two specific issues: the need of urine culture collection before all surgical treatment and the limitation of antibiotics use in everyday clinical practice. These acquisition are true not only for urological surgery but also for other surgical specialties.

In the case of orthopedic prosthetic surgery, we have new acquisitions that highlight that the ABU preoperative antibiotic treatment did not show any benefit and cannot be recommended [15]. Sousa *et al.* showed, in a multicenter study on candidates for total hip or total knee arthroplasty, that ABU is a common finding among total joint arthroplasty candidates and that it even emerges as an independent risk factor for prosthetic joint infection [15]. However, preoperative antibiotic treatment did not show benefit, so postponing surgery or even treating patients with known ABU before surgery cannot be recommended [15]. Moreover, in the case of open-heart surgery, some authors stated that in the absence of symptoms of urinary tract infection, urinalysis or urine culture are not necessary and not cost beneficial in the preoperative evaluation of patients scheduled for open-heart surgery [16]. Furthermore, even if the ABU is frequent among kidney transplant patients during the first year post transplantation, recent evidence showed no benefit for the antibiotic treatment of ABU in the short- and long-term follow-ups [17].

### 2.2. The Role of Asymptomatic Bacteriuria in Women Affected by Recurrent Urinary Tract Infections: To Treat or Not To Treat?

Recently, Cai *et al.* showed, in a randomized clinical trial, three important findings [12]:

(1) asymptomatic bacteriuria treatment is associated with a higher probability to develop symptomatic recurrence rate;
(2) asymptomatic bacteriuria treatment is associated with a modification of the isolated bacterial strains; and
(3) the presence of asymptomatic bacteriuria in patients affected by recurrent UTI, without any associated risk factor, has a protective role in development of subsequent symptomatic UTI, particularly when *Enterococcus faecalis* (*E. faecalis*) has been found.

This study was published in the *Clinical Infectious Diseases Journal* [12]. Here, we report the results of the study in line with the lecture performed by the author at the “Molecular UTI Conference—Urinary Tract Infection; Molecular Advances and Novel Therapies”, Scientific Director Prof. Catharina Svanborg (25–27 August 2014—Malmö; Sweden).

Cai *et al.* enrolled, in a randomized clinical trial study, between January 2005 and December 2009, all consecutively sexually active women patients with asymptomatic microbiologically demonstrated bacteriuria attending the same centre for history of recurrent UTIs [12]. All enrolled women underwent microbiological evaluation, even if they were asymptomatic, due to the routinely practice in their Sexually Transmitted Diseases Centre [12].

#### 2.2.1. Study Design, Schedule and Methodology

At the enrollment time, all women completed a baseline questionnaire about quality of life evaluation (an Italian version of the Quality of Well-Being, a validated, multi-attribute health scale) [12,18]. Then, they underwent urological examination, and provided two clean-catch midstream urine samples.

All women were randomized into two groups:

- Group A—Non-treated (only symptomatic episodes of urinary tract infection were recorded and treated; all treated patients were censored).
- Group B—Treated (all patients were treated with antibiotic therapy in accordance to antibiogram and on the basis of urologist’s choice; the most common used antibiotics were: fosfomycin-trometamol (31.4%) and nitrofurantoin (26.8%)).

All enrolled women were scheduled for follow-up visits and microbiological analysis at 3, 6 and 12 months from the enrolment. The principal measure of outcome was the recurrence-free rate at the end of the entire study period.

Cai *et al.* used the same definition for an episode of asymptomatic bacteriuria as Hooton, namely the presence of at least 10^5^ Colony forming Unit (CFUs) [5] of uropathogenic bacteria per milliliter in two consecutive voided urine specimens of a midstream urine specimen obtained from an asymptomatic woman on a routine scheduled visit [1,12]. All microbiological procedures have been performed in line with their previous studies [12,19,20].

#### 2.2.2. Clinical and Microbiological Findings

They screened 712 women with asymptomatic bacteriuria. Finally, 673 patients were analyzed:

- 312—Group A
- 361—Group B

#### 2.2.3. Baseline Results

The most common isolated pathogens at the baseline microbiological evaluation were *Escherichia coli* (*E. coli*) (38.4%,Group A; 39.3%, Group B) and *E. faecalis* (32.7%, Group A; 33.2%, Group B). No difference between the two groups at baseline evaluation in terms of clinical or microbiological findings have been found.

#### 2.2.4. At Three-Month Follow-Up Results

Three months after enrollment, 11 (3.5%) patients in Group A and 32 (8.8%) in Group B showed clinical symptoms related to UTI and then underwent specific antibiotic therapy and were censored. No significant difference was reported between the two groups (RR, 1.05; 95% CI, 1.01–1.10; *p* = 0.051).

#### 2.2.5. At Six-Month Follow-Up Results

Six months later, the patients in Group B (treated) showed a higher rate of symptomatic recurrences than those in Group A (not treated) (RR, 1.31; 95% CI, 1.21–1.42; *p* < 0.0001). The same statistically significant difference has also been reported in terms of quality of life questionnaire results (*t* = 86.37; df = 628; SE = 0.003; *p* < 0.001).

#### 2.2.6. At 12-Month Follow-Up Results (The End of the Study)

Twelve months after enrolment, the two Groups were different both in terms of recurrence rate (RR, 3.17; 95% CI, 2.55–3.90; *p* < 0.0001) and quality of life questionnaire results (*t* = 134.20; df = 507; SE = 0.002; *p* < .001). Moreover, no statistically significant difference has been reported between the two groups in terms of upper urinary tract infections rate. Furthermore, the Kaplan–Meier curve analysis showed that Group B had a higher probability of recurrence in comparison with Group A (RR, 2.14; SE = 0.187; *p* = 0.003).

Moreover, the most important finding is that the use of antimicrobial therapy is an independent factor affecting the risk of develop a symptomatic UTI in young sexually active women with history of recurrent UTI.

Another important finding to highlight is that, at the second and third follow-up evaluations, the majority of patients who were recurrence-free were found to have asymptomatic bacteriuria with *E. faecalis*.

## 3. Final Considerations and Take Home Messages

The evaluation of the role of ABU in pre-operative prophylaxis and in women affected by rUTIs is a key point in order to optimize the antibiotic usage and to prevent increased rate of resistant bacteria. The change in the isolated bacteria from the urinary tract after antibiotic therapy are well known and known to be dangerous in several cases. Beerepoot *et al.* demonstrated that oral administration of low dose antibiotics for the prevention of UTIs could cause ecological disturbances in normal intestinal microflora, while promoting the emergence of antimicrobial-resistant strains [21]. Several authors demonstrated that antibiotic therapy is able to disturb the ecological balance in the colon tract and to suppress the normal microflora [21,22]. The ecological effects of antibacterial agents on the human microflora should be the main reason of the negative effect of antibiotic therapy in the women affected by rUTIs with asymptomatic bacteriuria. The normal bacterial intestinal flora represents an extremely important defense mechanism, which effectively interferes with the establishment of many important enteric pathogens [23]. It is well known that mechanisms by which microorganisms suppress the growth of other microorganisms include modification of bile acids, stimulation of peristalsis, induction of immunologic responses, depletion of essential substrates from the environment, competition for attachment sites, creation of restrictive physiologic environments, and elaboration of antibiotic-like substances [22,23]. For example, has been demonstrated that normal bacterial intestinal flora is able to stimulate the production of secretory IgA, an antibody class unique to the mucosae [24]. In this sense, the presence of IgA in the intestinal lumen should be considered a primitive front line defense against induction of autoimmunity and invasion by microbial pathogens [25]. Components of the intestinal microbial flora also interact synergistically in the induction of disease or the utilization of substrate. In this sense, we can hypothesize that *E. faecalis* should be an extremely important defense mechanism, which effectively interferes with the establishment of many important enteric pathogens, such as *E. coli* [12].

### Take Home Messages

Please do not treat ABU in women affected by recurrent UTIs and improve your adherence to European Association of Urology guidelines on antibiotic prophylaxis in order to reduce related costs and the development of resistant bacteria.

## 4. Conclusions

In conclusion, nowadays, this is the time to think over our practice and change our way of thinking in the management of UTIs. Moreover, in patients affected by rUTIs, ABU can even have a protective role in preventing symptomatic recurrence, particularly when *Enterococcus faecalis* (*E. faecalis*) has been isolated.
